# High‐Sensitivity Cardiac Troponin T in Healthy Sport‐Participating Youth Aged 8–16 Years: Reference Values From the Cor‐School Cohort

**DOI:** 10.1002/ajhb.70176

**Published:** 2025-11-29

**Authors:** Saül Aixa‐Requena, Enric Conesa‐Milian, Vicenç Hernández‐González, Juan José Puente‐Lanzarote, Isaac López‐Laval, Álvaro de Pano‐Rodríguez, Joaquín Reverter‐Masià

**Affiliations:** ^1^ Human Movement Research Group (RGHM) University of Lleida Lleida Spain; ^2^ Physical Education and Sport Section University of Lleida Lleida Spain; ^3^ Service of Biochemistry Lozano Blesa University Hospital Zaragoza Spain; ^4^ Section of Physical Education and Sports University of Zaragoza Zaragoza Spain

**Keywords:** biological age, cardiac biomarkers, cardiomyocytes, puberty, youth

## Abstract

**Objectives:**

High‐sensitivity cardiac troponin T (hs‐cTnT) is widely used in adult cardiology, yet pediatric reference values remain scarce. This study aimed to establish reference values for hs‐cTnT in healthy sport‐participating youth aged 8–16 years from Spain; to examine differences by age, sex, and pubertal stage; and to explore associations with anthropometry and physical activity.

**Methods:**

This cross‐sectional study used baseline data from the Cor‐School cohort, including 733 organized sport participants aged 8–16 years from northeastern Spain. Anthropometry, pubertal status, biological maturity, and PHV timing were assessed alongside hs‐cTnT and habitual physical activity. Percentiles were calculated from detectable hs‐cTnT concentrations below the 99.75th percentile. Group comparisons, correlations, and regression models examined developmental patterns.

**Results:**

Among 733 youth, 39% had detectable hs‐cTnT and 4.7% exceeded 14 ng/L. The 97.5th and 99th percentiles were 15.2 ng/L (90% CI: 12.4–16.9) and 19.1 ng/L (90% CI: 15.2–21.3), peaking in early adolescence (11–13 years) and around PHV. Tanner Stage 4 showed the highest 99th percentile (20.4 ng/L), while Tanner 5 had lower values. Boys presented greater detectability (42% vs. 33%) and higher percentiles (99th: 19.1 vs. 15.6 ng/L). hs‐cTnT correlated weakly with maturity offset (*ρ* = 0.183, *p* = 0.002) and inversely with body fat (*ρ* = −0.170, *p* = 0.040), but no independent predictors remained in multivariable models (*R* ^2^ adj = 0.033).

**Conclusions:**

hs‐cTnT increases physiologically during adolescence, reflecting biological maturation. Pediatric‐specific reference values by age and developmental stage are recommended to avoid misclassification. As all participants were regularly engaged in organized sport, the reference values obtained likely represent the upper end of physiological hs‐cTnT concentrations in physically active youth.

## 1 Introduction

Cardiac biomarkers are biological molecules released into the bloodstream in response to myocardial stress or injury, providing essential diagnostic and prognostic information in the management of cardiovascular disease (Singh et al. 2010). Among these, cardiac troponins—specifically troponin T (cTnT) and troponin I (cTnI)—are recognized as the most specific and sensitive indicators of myocardial injury due to their exclusive expression in cardiac muscle tissue and their ability to detect even minimal cellular damage (Apple and Murakami 2005). Although traditionally associated with irreversible myocardial necrosis, emerging evidence suggests that troponin may also be released through nonclassical secretion mechanisms—such as vesicle‐mediated pathways or transient increases in membrane permeability—even in the absence of acute infarction (Gonzalez‐Rayas et al. 2022).

High‐sensitivity cardiac troponin T (hs‐cTnT) assays have enhanced the clinical utility of these biomarkers by enabling the detection of extremely low concentrations in healthy individuals and in subclinical conditions. They are now widely applied in both acute and chronic cardiovascular care and increasingly in population‐based screening and risk stratification (Clerico et al. 2022; Conesa‐Milian et al. 2023; Hammarsten et al. 2022). In adults, values above 14 ng/L—representing the 99th percentile of a healthy reference population—are used to diagnose myocardial infarction and predict adverse cardiovascular outcomes (McEvoy et al. 2023; Panotopoulos et al. 2022).

However, interpreting hs‐cTnT values in children and adolescents remains challenging. Adult thresholds may not be appropriate for pediatric populations due to physiological differences in cardiac morphology, hormonal regulation, and metabolic rate (Clerico et al. 2022). Baseline hs‐cTnT levels are typically lower in children and progressively increase during adolescence, possibly influenced by sex and pubertal development (Dong et al. 2024; Kiess et al. 2023; Lam et al. 2021). Although both hs‐cTnT and hs‐cTnI are used clinically, their expression profiles and assay‐specific characteristics differ in pediatric samples and require separate reference data (Bohn et al. 2019; Clerico et al. 2022).

This challenge aligns with broader principles in human biology, where biomarkers in childhood and adolescence are interpreted within the broader context of growth, maturation, and ecological influences rather than solely as indicators of disease. Extensive work in the field has shown that physiological markers often reflect developmental trajectories, life‐history trade‐offs, and biocultural environments (Bogin et al. 2007; Kuzawa and Bragg 2012; Modabbernia et al. 2021). Adolescence is characterized by pronounced endocrine and cardiovascular remodeling, including increases in cardiac mass and changes in metabolic and hormonal activity that influence circulating biomarkers (Dong et al. 2024; Floegel et al. 2025; Modabbernia et al. 2021; Robinson et al. 2023). Positioning hs‐cTnT within this framework underscores the need for reference values that account for sex‐, age‐, and maturation‐related variation, aligning with the core human‐biology aim of documenting physiological diversity across development.

Several studies have proposed pediatric reference intervals for hs‐cTnT, but many are limited by small sample sizes (e.g., < 100 participants) (Ameh and Brady 2024; Nlemadim et al. 2021), or by broad age ranges that span infancy to adolescence (Cai et al. 2024). Large population‐based datasets such as NHANES (USA) have included over 4000 healthy children and adolescents to define troponin thresholds (McEvoy et al. 2023), although results were not stratified by maturational status. In Europe, the LIFE Child study from Germany analyzed over 2500 individuals across the same broad age span (Kiess et al. 2022, 2023), highlighting age‐related increases but without detailed adjustment for maturation, physical activity, or sport participation. Similarly, large Chinese cohorts have reported data in nearly 1800 participants aged 0–18 years (Cai et al. 2024; Dong et al. 2024), leaving a gap for normative values from Mediterranean or Southern European youth. However, in all these cases the inclusion of very wide age ranges (e.g., pooling children from 1 to 18 years or from 10 to 18 years into single groups) results in heterogeneous distributions and reduced precision for adolescence‐specific reference values. This underscores the need for stratified, age‐specific analyses focused on narrower developmental windows.

This lack of age‐specific normative data is clinically significant. Even modest elevations in resting hs‐cTnT may reflect early myocardial stress, altered cardiac remodeling, or subclinical cardiovascular abnormalities in otherwise healthy youth (Conesa‐Milian et al. 2023; Dong et al. 2024; Magnetta et al. 2024). Establishing robust pediatric reference values stratified by age and sex is therefore essential for accurate interpretation and clinical application. These values could ultimately support early risk detection, personalized monitoring, and improved decision‐making in pediatric care.

To address this gap, the Cor‐School project investigates cardiovascular health in a large cohort of healthy school‐aged children and adolescents in northeastern Spain (Aixa‐Requena et al. 2024). This study presents first‐year results focused on defining sex‐, age‐, and maturity‐stratified reference values for hs‐cTnT, incorporating both self‐reported Tanner stage and the Moore–McKay maturity‐offset equation. In addition, we examined associations with anthropometric indicators such as body fat percentage, and daily physical activity, highlighting how body composition and lifestyle may influence baseline hs‐cTnT levels, an aspect rarely considered in previous pediatric cohorts.

## 2 Materials and Methods

### 2.1 Study Design and Participants

This cross‐sectional analysis is part of the first year of the Cor‐School project, an ongoing observational study investigating cardiovascular health in school‐aged children and adolescents in northeastern Spain (Aixa‐Requena et al. 2024). The study was conducted following the STROBE guidelines (von Elm et al. 2007).

A total of 733 children and adolescents (mean age: 11.7 ± 1.7 years), aged between 8 and 16 years, engaged in organized sport (school or club) were recruited from the northeastern region of Spain, specifically in southern Catalonia and northern Valencia, encompassing both urban and rural settings. The final sample comprised 296 girls and 437 boys, all of whom completed the assessment protocol. Given the recruitment strategy, the study sample represents children and adolescents engaged in organized sports rather than the general school population.

An invitation letter was distributed to all extracurricular sports programs of the area, and those that responded positively were included in the study. Participants and their legal guardians were informed of the study's objectives, procedures, potential risks, and benefits. Informed consent was obtained from both participants and guardians prior to data collection. A detailed medical history form was completed to screen for health conditions and assess eligibility. Participants were informed of their right to withdraw at any time without providing justification, with the assurance that their data would be permanently deleted upon withdrawal.

Before participation, parents or legal guardians completed a standardized cardiac health questionnaire based on the American Heart Association (AHA) 14‐element screening recommendations for cardiovascular evaluation of young athletes (Maron et al. 2014). The questionnaire included items regarding chest pain or discomfort during exercise, unexplained syncope or dizziness, excessive fatigue or shortness of breath with exertion, previous diagnosis of heart murmur, hypertension, myocarditis, or other cardiac disease, any physician‐imposed restriction on physical activity, and family history of premature sudden cardiac death (< 50 years), cardiomyopathy, long‐QT syndrome, or other inherited cardiac disorders. This standardized parental report, together with the PAR‐Q (Rodríguez 1994), was used to identify potential contraindications or cardiovascular risk factors before testing. Only children and adolescents without known cardiac conditions or reported symptoms were included in the study. This approach aligns with current European and international recommendations, which state that personal and family medical history, together with physical examination, constitute the fundamental components of preparticipation cardiovascular evaluation in youth, while ECG screening may be added depending on resources and national policies (Löllgen et al. 2015; Petek and Baggish 2020).

Exclusion criteria included uncontrolled asthma or chronic respiratory disease, congenital or acquired cardiovascular conditions (e.g., arrhythmia, cardiomyopathy), insulin‐dependent diabetes mellitus or other uncontrolled metabolic disorders, musculoskeletal injuries or chronic orthopedic conditions limiting physical activity, neurological disorders affecting motor function, and any physician‐imposed restriction on moderate‐to‐vigorous physical activity.

The procedures of this study have been approved by the Ethical Committee for Clinical Research of the Sports Administration of Catalonia (30/CEICGC/2020, approval date: December 15, 2020) and comply with the principles and recommendations of the latest revision of the Declaration of Helsinki (World Medical Association 2013).

### 2.2 Outcomes

#### 2.2.1 Anthropometric, Physical Activity and Maturity Assessments

Anthropometric assessments followed the standardized protocols of the International Society for the Advancement of Kinanthropometry (ISAK) (Esparza‐Ros et al. 2019). Body mass was measured using a Tanita MC‐780MA bioelectrical impedance analyzer (Tanita Corporation, Tokyo, Japan; ±0.1 kg), which also provided body fat percentage estimates. The Tanita MC‐780MA is widely used and considered reliable for assessing body composition in children and adolescents (Azoulay et al. 2021; Rusek et al. 2021; Salton et al. 2022; Verney et al. 2016). Height was measured with a portable stadiometer (Seca 213, Hamburg, Germany; ±0.1 cm). Body mass index (BMI) was calculated as weight (kg) divided by height squared (m^2^) and analyzed as a continuous variable to avoid potential misclassification. All measurements were performed by trained technicians in accordance with ISAK guidelines (Esparza‐Ros et al. 2019).

Daily physical activity was assessed using the Spanish version of the Physical Activity Questionnaire (PAQ), previously tested and validated for children (PAQ‐C) (Manchola‐González et al. 2017) and adolescents (PAQ‐A) (Martínez‐Gómez et al. 2009). These instruments provide a summary score reflecting habitual physical activity over the previous 7 days. The PAQ questionnaires have demonstrated acceptable reliability and validity in youth populations when administered under appropriate conditions (Bervoets et al. 2014; Manchola‐González et al. 2017; Marasso et al. 2021; Martínez‐Gómez et al. 2009; Voss et al. 2017).

Pubertal status was assessed through a validated self‐report questionnaire based on Tanner staging, which included descriptive text and standardized illustrations (Carskadon and Acebo 1993; Tanner and Whitehouse 1976). Participants were instructed to identify their developmental stage by comparing their physical characteristics (breast/genital development and pubic hair) with the references (Carskadon and Acebo 1993; Tanner and Whitehouse 1976). Maturity offset, defined as the estimated number of years from peak height velocity (PHV), was calculated using sex‐specific equations based on age and anthropometric measures (Moore et al. 2015).

#### 2.2.2 Blood Sampling and Biochemical Analysis

Venous blood samples (5 mL) were collected from an antecubital vein by qualified healthcare personnel—a specialized nurse contracted for this purpose. Samples were centrifuged at 3500 rpm for 10 min using a Sigma 2K‐15 centrifuge (Sigma Laborzentrifugen GmbH, Osterode am Harz, Germany). Serum aliquots were immediately frozen and stored at −80°C until analysis.

hs‐cTnT was measured using the Troponin T hs STAT electrochemiluminescence immunoassay on a Cobas E 601 analyzer (Roche Diagnostics, Penzberg, Germany). The analytical range is 3 to 10 000 ng/L, with an intra‐assay coefficient of variation of < 10% at a mean concentration of 13.5 ng/L. The upper reference limit (99th percentile) for hs‐cTnT in healthy adults is 14 ng/L (Giannitsis et al. 2010).

### 2.3 Statistical Analysis

All statistical analyses were conducted using JASP (version 0.18.1) (JASP Team 2023) and Microsoft Excel (version 16.66.1) for MacOs Catalina. The normality of continuous variables was assessed using the Shapiro–Wilk test and visual inspection of histograms and Q–Q plots. Descriptive statistics are reported as means ± standard deviations (SDs) or medians with interquartile ranges (IQRs), depending on distribution.

Values of hs‐cTnT below the assay's detection limit (3 ng/L) were treated as nondetectable, with no imputation applied. Cases with missing data were excluded pairwise. Associations between hs‐cTnT concentrations and continuous variables (e.g., age, body fat, maturity offset, PAQ score) were first examined using Spearman's rank correlation coefficient, given the nonnormal distribution of hs‐cTnT values. To evaluate the independence of these associations, a multiple linear regression model was performed including all predictors simultaneously, with adjusted *R* ^2^ reported to quantify the explained variance. Comparisons of hs‐cTnT concentrations were conducted between sexes (independent *t* tests or Mann–Whitney *U* tests), and across Tanner stages and maturity offset groups using one‐way ANOVA or Kruskal–Wallis tests, based on data normality.

Maturity offset was estimated using the Moore et al. (2015) equation (Moore et al. 2015). Participants were then classified relative to PHV into three groups using ±1‐year thresholds: pre‐PHV (≥ 1 year before PHV), circa‐PHV (within ±1 year of PHV), and post‐PHV (≥ 1 year after PHV). This windowing approach is consistent with prior maturation‐based classifications in youth sport research and practice (Cumming et al. 2017).

The Levene's test was used to assess homogeneity of variances prior to parametric testing. When significant differences were found, pairwise comparisons were conducted using Dunn's post hoc test with Bonferroni correction to control for family‐wise error rate. Effect sizes for sex comparisons were reported using the rank‐biserial correlation (*r*(*b*)). While not originally described by Cohen (2013), interpretation followed common benchmarks for correlation coefficients: small (0.10), medium (0.30), and large (0.50), as suggested in applied literature (Kerby 2014).

In the case of ordinal variables (e.g., Tanner stage), group differences were additionally assessed using Pearson's *χ* ^2^ test, and effect sizes were expressed as Cramér's *V*. Interpretation of Cramér's *V* followed widely accepted thresholds for degrees of freedom (df = *k* − 1): small (*V* = 0.1), medium (*V* = 0.3), and large (*V* ≥ 0.5), in accordance with Cohen's criteria (Cohen 2013).

Percentile values (2.5th, 10th, 25th, 50th, 75th, 90th, 97.5th, and 99th) of hs‐cTnT were calculated for the total sample and stratified by sex and age group to provide clinically useful normative reference data. For the 97.5th and 99th percentiles, 90% confidence intervals were estimated using nonparametric bootstrap resampling.

Two extreme outliers were excluded before analysis. Candidate outliers were first identified by visual inspection of scatter and box plots and subsequently verified against the sex‐specific 99.75th percentile threshold, following the approach applied in previous pediatric troponin studies from the LIFE Child cohort (Kiess et al. 2022, 2023). Similar exclusion criteria based on the 0.25th–99.75th percentile range have been applied in large biomedical datasets, including studies of blood pressure and nutritional biomarkers in hemodialysis populations (Kalantar‐Zadeh et al. 2005; Lertdumrongluk et al. 2020; Park et al. 2013; Shinaberger et al. 2006), as well as in laboratory and metabolomic analyses (Jia et al. 2018). This conservative method ensures that only values highly unlikely to represent physiological concentrations are removed.

A post hoc power analysis was conducted using G*Power (version 3.1.9.6) to evaluate whether the available sample size was adequate for detecting meaningful differences between groups. Assuming a small‐to‐moderate effect size (Cohen's *d* = 0.3; *f* = 0.25) and an alpha level of 0.05, the analysis showed a statistical power greater than 0.99 for comparisons by sex (*n* = 437 vs. 296), age group (3 groups), and Tanner stage (5 groups). These results confirm that the sample was sufficient to detect relevant group differences with high confidence.

A two‐tailed *p* value < 0.05 was considered statistically significant for all analyses.

## 3 Results

### 3.1 Descriptive Characteristics and Sex Differences

Descriptive data for the overall sample and stratified by sex are shown in Table 1. Boys and girls were similar in age (median 12 years in both groups) and height (both 153 cm), with no significant differences. Weight and BMI also did not differ significantly between sexes. Girls presented a significantly higher body fat percentage compared to boys (24.8% ± 7.4% vs. 19.9% ± 7.6%, *p* < 0.001), and were more advanced in pubertal development, as reflected by higher Tanner stages and maturity offset, closer to PHV (−0.2 ± 1.5 vs. –2.1 ± 2.2 years, *p* < 0.001). In addition, girls reported lower PAQ scores compared to boys (2.9 ± 0.6 vs. 3.1 ± 0.6, *p* < 0.001), suggesting lower levels of self‐reported physical activity.

**TABLE 1:** Descriptive data of participants overall and by sex.

	*n*	Overall	*n*	Boys	*n*	Girls	*p*	Effect size
Age (years)	733	12 (2)	437	12 (3)	296	12 (2)	0.807	0.043
Height (cm)	730	153 ± 12	436	153 (19)	294	153 (15)	0.946	0.044
Weight (kg)	730	45.7 (17.5)	436	45.6 (18.3)	294	46.3 (15.1)	0.121	0.044
BMI (kg/m^2^)	730	19.4 (4.1)	436	19.1 (4.1)	294	19.6 (4.2)	0.065	0.044
Body fat (%)	453	22.1 (7.9)	253	19.9 (7.6)	200	24.8 (7.4)	< 0.001***	0.055
Pubertal stage (Tanner 1–5)	664	115/216 / 183/128/22	396	77/141/100/67/11	268	38/75/83/61/11	0.027***	0.129
Maturity offset (years)	730	−1.3 (2.5)	436	−2.1 (2.2)	294	−0.2 ± 1.5	< 0.001***	0.044
PAQ score (1–5)	621	3.0 ± 0.6	372	3.1 ± 0.6	249	2.9 ± 0.6	< 0.001***	0.403

*Note:* Values are presented as median (IQR) or mean ± SD, depending on the distribution. Effect sizes for continuous variables are reported as rank biserial correlation. For ordinal variables such as Tanner stage, values are reported as frequencies per stage, and effect size is provided using Cramér's *V*. *p*‐values were obtained using Mann–Whitney *U* test (for sex comparisons) or *χ* ^2^ test (for categorical comparisons).

Abbreviations: BMI = body mass index; hs‐cTnT = high‐sensitivity cardiac troponin T.

*** *p* < 0.001.

### 3.2 Group Differences by PHV and Tanner Stage

As expected, anthropometric and developmental variables increased progressively across maturity groups, both when classified by PHV status and by Tanner stage (all *p* < 0.001). In PHV groups, post‐PHV participants showed higher body fat percentage and lower PAQ scores compared to pre‐ and circa‐PHV peers. Similarly, across Tanner stages, age, height, weight, BMI, and maturity offset increased consistently, while body fat percentage remained stable. A small but significant difference in PAQ scores was observed between Tanner stages 2 and 4 (*p* = 0.043). Detailed descriptive data by PHV group and Tanner stage are presented in Supplementary Tables S1 and S2.

### 3.3 Hs‐cTnT Detection and Concentrations

**FIGURE 1:**
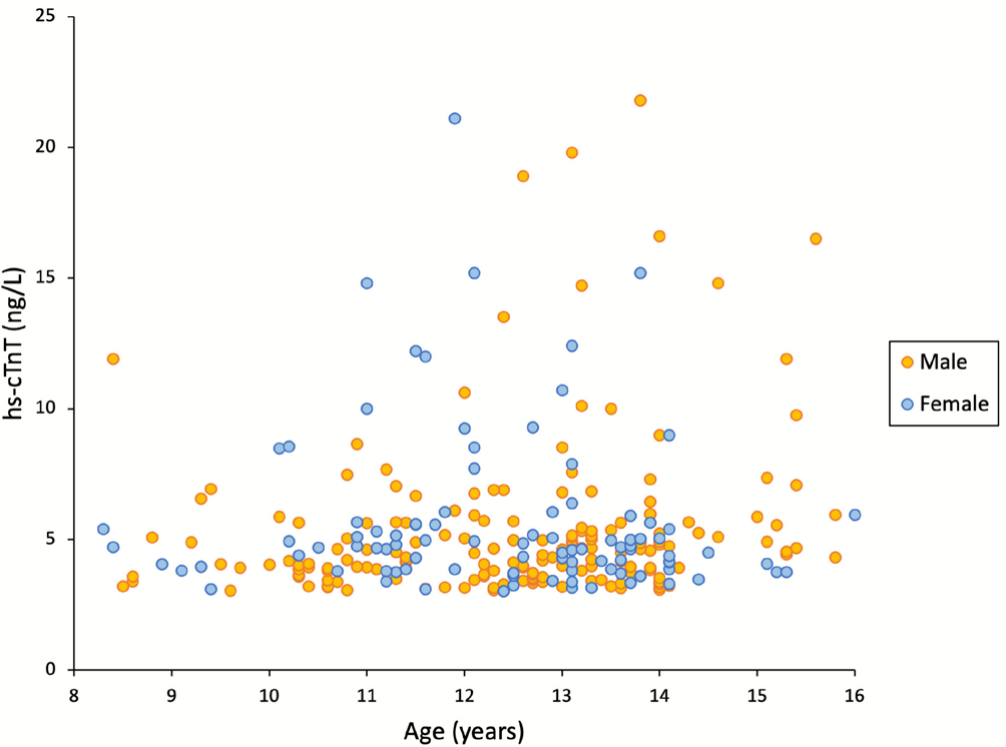
Scatterplot of baseline hs‐cTnT concentrations by age and sex.

Hs‐cTnT was detectable in 39% of participants, while 61% had concentrations below the limit of detection (< 3 ng/L) (Table 2). Detectability was slightly higher in boys (42%) than in girls (33%), but the difference was not statistically significant (*p* = 0.239). Overall, 4.7% of detectable participants exceeded the 14 ng/L threshold. Figure 1 shows the scatterplot of hs‐cTnT concentrations by age and sex, illustrating the wide dispersion of detectable values.

**TABLE 2:** hs‐cTnT detection and concentrations by sex, maturation phase and pubertal stage.

Group	*n*	*n* (%) Detectable (≥ 3 ng/L LoD)	> 14 ng/L, *n* (%)	hs‐cTnT (ng/L)	*p*	Differences between
Overall	718	278 (39%)	13 (4.7%)	4.6 (1.8)	NA	NA
Sex^a^						
Male	429	182 (42%)	8 (4.4%)	4.5 (1.9)	0.239	NA
Female	289	96 (33%)	5 (5.2%)	4.7 (1.8)		
Maturation phase^b^						
Pre‐PHV	400	132 (33%)	3 (2.3%)	4.1 (1.6)	< 0.001***	^d^, ^e^
Circa‐PHV	238	108 (45%)	10 (9.3%)	5.0 (2.6)		
Post‐PHV	80	38 (48%)	0 (0%)	4.2 (1.1)		
Pubertal stage^c^						
Tanner 1	111	38 (34%)	1 (2.6%)	3.9 (1.5)	0.488	NA
Tanner 2	211	73 (35%)	4 (5.5%)	4.9 (1.9)		
Tanner 3	180	65 (36%)	4 (6.2%)	4.6 (1.9)		
Tanner 4	125	70 (56%)	4 (5.7%)	4.7 (1.9)		
Tanner 5	22	8 (36%)	0 (0%)	5.4 (2.6)		

*Note:* Values of hs‐cTnT are presented as median (IQR).

Abbreviation: NA = not applicable.

*** *p* < 0.001.

^a^ Sex differences were assessed using the Mann–Whitney *U* test due to the nonnormal distribution of the data.

^b^ Group differences were assessed using the Kruskal–Wallis test; post hoc pairwise comparisons were conducted using Dunn's test with Bonferroni correction.

^c^ Group differences were assessed using the chi square test.

^d^ Statistically significant pairwise differences: pre‐PHV versus circa‐PHV.

^e^ Statistically significant pairwise differences: circa‐PHV versus post‐PHV.^1^

When stratified by maturation phase, detectability increased from 33% in pre‐PHV participants to 45% in circa‐PHV and 48% in post‐PHV adolescents (*p* < 0.001). Pairwise comparisons confirmed significant differences between pre‐ and circa‐PHV, as well as between circa‐ and post‐PHV groups. Median concentrations also rose from 4.1 ng/L in pre‐PHV to 5.0 ng/L in circa‐PHV before declining to 4.2 ng/L in post‐PHV.

Across Tanner stages, the proportion of detectable values ranged between 34% and 56%, with the highest prevalence in Tanner stage 4. Median hs‐cTnT concentrations remained relatively stable (3.9–5.4 ng/L), and overall differences were not significant (*p* = 0.488).

### 3.4 Hs‐cTnT Percentile Distributions

Percentile values are presented in Table 3. In the total sample, the 97.5th and 99th percentiles reached 15.2 ng/L (90% CI: 12.4–16.9) and 19.1 ng/L (90% CI: 15.2–21.3), respectively, exceeding the conventional adult cutoff of 14 ng/L. In boys and girls, the 99th percentiles were 19.1 and 15.6 ng/L, respectively.

**TABLE 3:** Hs‐cTnT percentiles by sex, age, maturation phase and pubertal stage.

	Detectable, *n* (%)	> 14 ng/L, *n* (%)	2.5th	10th	25th	50th	75th	90th	97.5th [90% CI]	99th [90% CI]
Overall	278 (39%)	13 (4.7%)	3.1	3.3	3.8	4.6	5.6	8.6	15.2 [12.4–16.9]	19.1 [15.2–21.3]
Sex										
Male	182 (42%)	8 (4.4%)	3.1	3.2	3.7	4.5	5.6	7.6	15.7 [11.9–18.9]	19.1 [15.1–21.8]
Female	96 (33%)	5 (5.2%)	3.1	3.4	3.9	4.7	5.6	9.3	15.1 [11.5–19.0]	15.6 [12.6–21.1]
Age										
8.0–8.9	8 (24%)	0 (0%)	3.2	3.3	3.5	4.4	5.2	7.3	10.8 [5–11.9]	11.4 [5.1–11.9]
9.0–9.9	9 (28%)	0 (0%)	3.1	3.1	3.8	4.0	4.9	6.6	6.8 [4.7–6.9]	6.9 [4.8–6.9]
10.0–10.9	34 (31%)	0 (0%)	3.1	3.2	3.7	4.0	5.0	7.0	8.6 [6.3–8.6]	8.6 [7.5–8.6]
11.0–11.9	42 (32%)	2 (5%)	3.1	3.5	3.9	4.8	5.6	9.8	14.7 [9.9–21.1]	18.5 [12–21.1]
12.0–12.9	58 (36%)	4 (7%)	3.1	3.3	3.5	4.2	5.7	8.9	14.6 [9.3–18.9]	16.9 [11.2–18.9]
13.0–13.9	82 (51%)	4 (5%)	3.2	3.4	4.0	4.7	5.6	8.5	15.2 [10.1–21.7]	20.2 [12.8–21.8]
14.0–14.9	27 (50%)	2 (7%)	3.1	3.3	3.5	4.5	5.2	9.0	15.4 [8.7–16.6]	16.1 [9–16.6]
15.0–15.9	16 (59%)	1 (6%)	3.7	3.9	4.4	5.2	7.2	10.8	14.8 [8.7–16.5]	15.8 [9.4–16.5]
16.0–16.9	2 (40%)	0 (0%)	4.1	4.3	4.5	5.0	5.5	5.7	5.9 [NA]	5.9 [NA]
Maturation phase										
Pre‐PHV	132 (33%)	3 (2.3%)	3.1	3.2	3.6	4.1	5.2	6.9	10.2 [7.6–14.7]	13.9 [8.9–18.9]
Circa‐PHV	108 (45%)	10 (9.3%)	3.2	3.5	4.2	5.0	6.8	12.3	17.7 [14.9–21.3]	21.0 [16.4–21.8]
Post‐PHV	38 (48%)	0 (0%)	3.2	3.4	3.8	4.3	4.9	7.2	9.0 [7.2–9.8]	9.5 [7.4–9.8]
Pubertal stage										
Tanner 1	38 (34%)	1 (2.6%)	3.0	3.1	3.4	3.9	4.9	6.7	9.3 [6.7–18.9]	15.1 [6.8–18.9]
Tanner 2	73 (35%)	4 (5.5%)	3.2	3.5	4.0	4.9	5.9	11.7	14.9 [12.0–21.1]	16.9 [12.9–21.1]
Tanner 3	65 (36%)	4 (6.2%)	3.1	3.4	3.8	4.6	5.6	9.0	15.0 [9.5–16.6]	15.7 [10.0–16.6]
Tanner 4	70 (56%)	4 (5.7%)	3.1	3.4	3.9	4.7	5.8	8.0	17.5 [10.0–21.8]	20.4 [13.5–21.8]
Tanner 5	8 (36%)	0 (0%)	3.2	3.6	4.4	5.4	7.0	7.5	7.6 [6.7–7.7]	7.7 [6.8–7.7]

Stratification by age groups showed a progressive increase in detection rates and upper percentiles with advancing age. Detection rates increased from 24% to 32% in participants aged 8–11 years to 36%–59% in those aged 12–15 years. Notably, 51% of 13‐year‐olds and 59% of 15‐year‐olds had detectable values. The 99th percentile rose from 8.6 ng/L at age 10 to 20.2 ng/L at age 13 and 15.8 ng/L at age 15. The highest 97.5th and 99th percentiles were observed between ages 11 and 13 years, indicating that early adolescence may represent a phase of greater variability in basal hs‐cTnT concentrations.

By maturation phase, the 99th percentile peaked at 21.0 ng/L (90% CI: 16.4–21.8) in the circa‐PHV group, while post‐PHV participants displayed lower upper percentiles (9.5 ng/L). At the pubertal stage level, Tanner Stage 4 showed the highest 99th percentile (20.4 ng/L), whereas Tanner Stage 5 presented lower and narrower distributions, likely reflecting the smaller sample size. Overall, 4.7% of detectable participants exceeded the 14 ng/L threshold.

### 3.5 Correlation Analysis

Correlation and regression analyses are summarized in Table 4. In bivariate analyses, hs‐cTnT was positively associated with maturity offset (*ρ* = 0.183, *p* = 0.002) and negatively associated with body fat percentage (*ρ* = −0.170, *p* = 0.040). No significant associations were found with age or PAQ score.

**TABLE 4:** Associations between hs‐cTnT and developmental, anthropometric, and physical activity variables.

Predictor	*n*	*ρ* ^a^	*p* ^a^	*B* ^b^	*β* ^b^	*p* ^b^
Age	276	0.099	0.102	0.017	0.008	0.943
Maturity offset	276	0.183**	0.002	0.153	0.094	0.398
Body fat %	146	−0.170*	0.040	−0.072	−0.125	0.200
PAQ score	240	0.115	0.075	1.152	0.189	0.054
Model total^**b**^		NA	NA	NA	NA	*p* = 0.099 (*R* ^2^ adj = 0.033)

*Note:* Adjusted *R*² for the multiple model = 0.033; overall model *p* = 0.099. *p* < 0.05 was considered statistically significant.

Abbreviations: *β* = standardized coefficient; *B* = unstandardized coefficient.

^a^ Values correspond to bivariate associations (Spearman's *ρ*).

^b^ Values correspond to coefficients from the multiple linear regression including all predictors simultaneously.

* *p* < 0.05.

** *p* < 0.01.

In the multiple linear regression including all predictors simultaneously, none remained significant (all *p* ≥ 0.054). The overall model was not significant (*p* = 0.099) and explained only 3.3% of the variance in baseline hs‐cTnT, indicating that the observed associations were not independent and that developmental, anthropometric, and physical activity factors account for only a very small proportion of variability.

## 4 Discussion

This study provides novel reference data for hs‐cTnT in Spanish children and adolescents aged 8–16 years, all engaged in organized sport. We report that approximately 39% of participants presented detectable hs‐cTnT, and 4.7% exceeded the conventional adult diagnostic cutoff of 14 ng/L. Percentile analysis showed that the 97.5th and 99th percentiles reached 15.2 and 19.1 ng/L, respectively, with peaks around early adolescence and the circa‐PHV phase. Importantly, this is the first study to stratify reference values by maturity offset and PHV, providing a developmental perspective beyond age and Tanner stage. These findings highlight the need for age‐ and maturity‐specific interpretation of hs‐cTnT during adolescence.

### 4.1 Age, Maturity, and Sex Effects

Detection rates and upper percentiles increased progressively with age, from 24% to 32% in participants aged 8–11 years to over 50% in those aged 13–15 years. The highest 99th percentiles were observed between 11 and 16 years, coinciding with the circa‐PHV stage, where values exceeded 20 ng/L. This concentration of high percentiles around PHV aligns with prior evidence from the NHANES, CALIPER and LIFE Child cohorts, which also showed age‐ and maturity‐related increases in boys (Bohn et al. 2019; Fang et al. 2024; Kiess et al. 2022, 2023; Lam et al. 2021; McEvoy et al. 2023). The use of the Moore–McKay maturity‐offset equation (Moore et al. 2015) allowed us to complement self‐reported Tanner staging, which is prone to misclassification, especially in younger children and in Tanner Stage 5, where our sample was small. By combining both measures, we identified a pattern of increasing hs‐cTnT concentrations through adolescence up to the period around PHV, with some decline thereafter, consistent with a maturation‐linked effect rather than a purely monotonic rise.

Sex differences were minimal. Boys had slightly higher detectability rates, but 97.5th percentiles were similar between sexes. This matches previous reports showing that apparent sex differences are largely explained by earlier female maturation rather than intrinsic biological factors (Cao et al. 2024; Fang et al. 2024).

Some mechanistic hypotheses point to sex hormone effects: males generally develop greater myocardial mass, and estrogens in females may exert protective effects on cardiomyocytes (Cao et al. 2024; Chaulin 2023). Additionally, the surge in testosterone in boys beginning around age 12 has been proposed to influence troponin dynamics, as suggested by preliminary analyses in adolescent cohorts (Fang et al. 2024; Greene et al. 2022) regarding distribution of high‐sensitivity troponin by sex. This hormonal transition could partly explain the higher hs‐cTnT values observed in participants at Tanner Stage IV, a period corresponding to rapid increases in testosterone and cardiac growth.

These results emphasize that maturity‐adjusted rather than sex‐specific thresholds may be more appropriate for pediatric populations.

### 4.2 Body Composition and Physical Activity

We observed weak inverse associations between hs‐cTnT and body fat percentage. This finding aligns with evidence that lean mass and markers of somatic growth are positively associated with hs‐cTnT concentrations in youth populations, rather than adiposity per se. For example, Fang et al. (2024) reported that lean mass index was a significant positive determinant of hs‐cTnT in US children and adolescents (Fang et al. 2024), while Kiess et al. (2022) found that body size and growth‐related markers influenced troponin concentrations in the LIFE Child cohort (Kiess et al. 2022, 2023).

Although the correlation with physical activity (PAQ score) did not reach significance, it is possible that isolated participants did not fully comply with pretest rest instructions, potentially influencing baseline values. Nonetheless, outliers were excluded, minimizing the effect on final estimates. Importantly, our cohort consisted of sport‐enrolled youth, a factor that may have elevated overall hs‐cTnT levels compared with general pediatric populations (Aengevaeren et al. 2021; Cirer‐Sastre et al. 2020; Conesa‐Milian et al. 2023).

Exercise is a well‐established trigger of transient troponin release in adolescents (Cirer‐Sastre et al. 2019; Conesa‐Milian et al. 2023; Tian et al. 2012), and recent reviews highlight the role of training load and cardiac remodeling as modulators of baseline variability (Chaulin 2021; Cirer‐Sastre et al. 2019, 2020; Conesa‐Milian et al. 2023; Gonzalez‐Rayas et al. 2022; Hammarsten et al. 2022).

### 4.3 Clinical Implications

Our results reinforce that adult diagnostic cutoffs are inappropriate for pediatric populations. In our cohort, 97.5th and 99th percentiles in early adolescence exceeded the adult threshold of 14 ng/L, a finding consistent with other pediatric cohorts (Kiess et al. 2022; Lam et al. 2021). Values above the adult 14 ng/L cutoff observed in 4.7% of participants should not be interpreted as indicative of myocardial injury. These concentrations likely represent physiological elevations associated with cardiac growth and remodeling during adolescence, rather than pathological processes. Without age‐ or maturity‐specific interpretation, healthy adolescents could be misclassified, leading to unnecessary investigations (Aakre et al. 2023; Fang et al. 2024; Lam et al. 2021; Velilla Moliner et al. 2017). Pediatric reference values should be based on developmental stage, with the 97.5th percentile increasingly recommended as a stable and clinically relevant cutoff (Aakre et al. 2023; McEvoy et al. 2023). The clinical interpretation of these findings is supported by recent large cohort studies demonstrating that careful use of hs‐cTnT does not lead to unnecessary diagnoses or procedures, but rather may improve efficiency and safety in patient management (McDermott et al. 2024; Nguyen et al. 2024; Ola et al. 2021). As the entire cohort consisted of sport‐enrolled children and adolescents, these percentiles may reflect the upper range of normal hs‐cTnT concentrations in physically active youth, and should not be directly extrapolated to sedentary or clinical populations.

Interpreting our findings through a human‐biology framework further emphasizes that biomarker variability during adolescence often reflects normative developmental processes. Prior work has documented that cardiac growth, pubertal endocrine activation, and shifts in metabolic and inflammatory regulation contribute to predictable changes in circulating biomarkers during this life stage (Dong et al. 2024; Floegel et al. 2025; Modabbernia et al. 2021; Robinson et al. 2023). Within this perspective, detectable or moderately elevated hs‐cTnT in healthy youth should be viewed as part of developmental physiology rather than evidence of subclinical pathology. Our sex‐, age‐, and maturity‐stratified percentiles therefore contribute to ongoing efforts in human biology to generate population‐specific reference standards that improve the interpretation of biological variation across adolescence.

### 4.4 Strengths, Limitations, and Future Directions

Key strengths include the large sample size, standardized blood collection, and the novel use of maturity offset to complement Tanner staging, providing a robust developmental framework. However, limitations must be acknowledged. Analyses were restricted to detectable values below the 99.75th percentile, as in previous reference studies (Kiess et al. 2022, 2023), reducing effective sample sizes for percentile estimation. Tanner staging was self‐reported, with a particularly small sample in Tanner 5, limiting confidence in late‐pubertal reference values. Furthermore, the exclusive inclusion of sport‐enrolled participants may bias reference intervals upward compared with general pediatric populations. Finally, the cross‐sectional design cannot establish within‐individual trajectories. Future longitudinal studies should integrate direct measures of training load and repeated hs‐cTnT sampling to better understand the biological drivers of troponin variability across adolescence.

Beyond these methodological considerations, the variability observed in basal hs‐cTnT concentrations—similar to that seen after exercise—cannot be fully explained by covariates such as sex, maturity, or cardiorespiratory fitness. This indicates the existence of interindividual differences in hs‐cTnT release within healthy youth, whose physiological basis and potential clinical relevance remain to be clarified. Future longitudinal and mechanistic studies should aim to elucidate these determinants to refine interpretation in both clinical and sport‐related contexts.

## 5 Conclusions

This study provides novel reference values for hs‐cTnT in healthy Spanish children and adolescents aged 8–16 years. We show that detectable concentrations increase in prevalence during early adolescence, peaking around the period of PHV, where the highest percentiles were observed. Importantly, the 97.5th and 99th percentiles in this age range often exceeded the conventional adult cutoff of 14 ng/L, underlining the inappropriateness of applying adult diagnostic thresholds to pediatric populations.

Sex differences were minimal and largely explained by differences in biological maturation rather than intrinsic sex‐specific factors. Moreover, weak associations with body composition and physical activity suggest that cardiac developmental processes, rather than lifestyle characteristics, are the primary drivers of troponin variability during growth.

The percentile tables may serve as a useful clinical reference when evaluating baseline hs‐cTnT in pediatric athletes, supporting the development of age‐ and maturity‐specific diagnostic frameworks.

Overall, our findings highlight the need for age‐ and maturity‐adjusted pediatric reference values for hs‐cTnT. These results contribute to refining the clinical interpretation of cardiac biomarkers in youth, reducing the risk of false positives and unnecessary investigations in healthy, developing individuals.

## Author Contributions

**Saül Aixa‐Requena:** conceptualization, formal analysis, investigation, data curation, writing – original draft preparation, visualization. **Enric Conesa‐Milian:** conceptualization, methodology, investigation, resources, writing – review and editing. **Vicenç Hernández‐González:** investigation, data curation, project administration. **Juan José Puente‐Lanzarote:** investigation, resources. **Isaac López‐Laval:** methodology, investigation, resources. **Álvaro de Pano‐Rodríguez:** investigation, resources. **Joaquín Reverter‐Masià:** conceptualization, methodology, writing – review and editing, supervision, project administration, funding acquisition. All authors have read and agreed to the published version of the manuscript.

## Funding

This research was funded by the State Program for Research, Development, and Innovation Oriented to the Challenges of Society, within the framework of the State Plan for R + D + I 2020–2024. The project title is as follows: “Assessment of various health parameters and physical activity levels in Primary and Secondary School” (Grant PID2020‐117932RB‐I00). Additionally, the research was supported by the consolidated research group Human Movement, Generalitat de Catalunya (021 SGR 01619). S.A.‐R. was supported by the predoctoral grants program FI SDUR from the Department of Research and Universities of the Generalitat de Catalunya, cofunded by the European Social Fund Plus (2024 FISDU 00122).

## Ethics Statement

The procedures of this project have been approved on December 15, 2020 by the Ethics Committee for Clinical Research of the Catalan Sports Council (30/CEICGC/2020) and comply with the principles and recommendations of the latest revision of the Declaration of Helsinki (World Medical Association 2013).

## Consent

Informed consent was obtained from all participants and their legal guardians prior to any data collection as part of the protocol procedures.

## Conflicts of Interest

The authors declare no conflicts of interest.

## Supporting information


**Table S1:** Descriptive data by PHV moment.
**Table S2:** Descriptive data by pubertal stage.

## Data Availability

The data and code supporting the findings of this study will be openly available in the Repositori de Dades de Recerca (Universitat de Lleida) upon publication. A persistent identifier (DOI) will be provided as soon as it is minted. In the interim, materials are available from the corresponding author on reasonable request.

## Endnotes

^1^
* *p* < 0.05. ** *p* < 0.01. *** *p* < 0.001.
