# Supplementary material for: High‐Sensitivity Cardiac Troponin T in Healthy Sport‐Participating Youth Aged 8–16 Years: Reference Values From the Cor‐School Cohort
Source: Am J Hum Biol. 2025 Nov 29;37(12):e70176. doi: 10.1002/ajhb.70176 (PMC12665097; doi:10.1002/ajhb.70176)
Supplement: Supplementary file 1 — Table S1: Descriptive data by PHV moment. Table S2: Descriptive data by pubertal stage. [file AJHB-37-e70176-s001.docx]

**High-Sensitivity Cardiac Troponin T in Healthy Sport-Participating Youth Aged 8 to 16 Years: Reference Values from the Cor-School Cohort**

Supplementary Materials

**Table S1**. Descriptive data by PHV moment

|  | n | Pre-PHV | n | Circa-PHV | n | Post-PHV | p | Differences between |
| --- | --- | --- | --- | --- | --- | --- | --- | --- |
| Age (years) | 405 | 11.3 (2.2) | 246 | 12.9 (1.5) | 82 | 14.1 (1.4) | <0.001*** | a b c |
| Height (cm) | 405 | 146 (15) | 243 | 158 ± 10 | 82 | 163 (10) | <0.001*** | a b c |
| Weight (kg) | 405 | 39.9 (14.1) | 243 | 50.9 (14.9) | 82 | 57.3 (13.3) | <0.001*** | a b c |
| BMI (kg/m^2^) | 405 | 18.5 (4.1) | 243 | 19.7 (4) | 82 | 20.8 (3.6) | <0.001*** | a b c |
| Body Fat (%) | 231 | 21.8 (8.35) | 164 | 21.7 (7.3) | 58 | 25.9 ± 6.0 | <0.001*** | b c |
| Pubertal Stage (Tanner 1-5) | 370 | 100 / 156 / 76 / 29 / 9 | 227 | 15 / 55 /82 / 66 / 9 | 67 | 0 / 5 / 25 / 33 / 4 | <0.001*** | NA |
| Maturity offset (years) | 405 | -2.5 (1.7) | 243 | -0.3 1.0) | 82 | 1.6 (0.6) | <0.001*** | a b c |
| PAQ Score (1-5) | 348 | 3.1 ± 0.6 | 204 | 3.0 ± 0.6 | 69 | 2.7 ± 0.6 | <0.001*** | b c |

BMI = body mass index;; NA = not applicable. Values are presented as median (IQR) or mean ± SD, depending on the distribution. Tanner stage values are presented as absolute frequencies for each pubertal stage, and their group differences were assessed using the chi-square test. Kruskal–Wallis test used to assess group differences. Post hoc pairwise comparisons conducted using Dunn’s test with Bonferroni correction. Statistically significant pairwise differences: a = pre-PHV vs. circa-PHV; b = circa-PHV vs. post-PHV; c = pre-PHV vs. post-PHV.

**Table S2**. Descriptive data by pubertal stage

|  | n | Tanner 1 | n | Tanner 2 | n | Tanner 3 | n | Tanner 4 | n | Tanner 5 | p | Differences between |
| --- | --- | --- | --- | --- | --- | --- | --- | --- | --- | --- | --- | --- |
| Age (years) | 115 | 10 (2.5) | 216 | 11 (2) | 183 | 12 (2) | 128 | 13 (1) | 22 | 12.5 ± 1.9 | < 0.001*** | a b c d e f g |
| Height (cm) | 114 | 142 ± 10 | 216 | 148 ± 9 | 183 | 158 ± 10 | 126 | 163 ± 9 | 22 | 161 ± 13 | < 0.001*** | a b c d e f g h |
| Weight (kg) | 114 | 36.3 (10.9) | 216 | 40.5 (13.8) | 183 | 49.4 (15.8) | 126 | 56.1 ± 10.3 | 22 | 54.9 ± 9.5 | < 0.001*** | a b c d e f g h |
| BMI (kg/m^2^) | 114 | 18.3 (3.2) | 216 | 18.4 (3.7) | 183 | 19.5 (3.7) | 126 | 20.7 (3.9) | 22 | 20.7 (2.8) | < 0.001*** | b c d e f g h i |
| Body Fat (%) | 66 | 22 (5.8) | 129 | 22.9 (7) | 112 | 22.1 (8.6) | 73 | 22.1 (11.4) | 13 | 22.5 ± 6.8 | 0.591 | NA |
| Maturity offset (years) | 114 | -2.9 (1.7) | 216 | -2.0 (2.1) | 183 | -0.6 (2.2) | 126 | -0.1 (1.9) | 22 | -0.6 ± 2.3 | < 0.001*** | a b c d e f g h |
| PAQ Score (1-5) | 105 | 3.0 ± 0.6 | 194 | 3.1 ± 0.6 | 172 | 3.0 ± 0.6 | 111 | 2.9 ± 0.6 | 19 | 3.0 ± 0.7 | 0.043* | f |

BMI = body mass index; hs-cTnT = high-sensitivity cardiac troponin T; NA = not applicable. Values are presented as median (IQR) or mean ± SD, depending on the distribution. Kruskal–Wallis test used to assess group differences. Post hoc pairwise comparisons conducted using Dunn’s test with Bonferroni correction. Statistically significant pairwise differences: a = stage 1 vs. 2; b = 1 vs. 3; c = 1 vs. 4; d = 1 vs. 5; e = 2 vs. 3; f = 2 vs. 4; g = 2 vs. 5; h = 3 vs. 4; i = 3 vs. 5; j = 4 vs. 5.
